# A multilevel analysis on the predictors of client satisfaction with family planning services in Ethiopia: evidence from the Ethiopian service provision assessment (ESPA) 2021/22

**DOI:** 10.1080/16549716.2025.2463215

**Published:** 2025-02-13

**Authors:** Michael Endale Mengesha, Henrik Holmberg

**Affiliations:** Department of Epidemiology and Global Health, Umeå University, Umeå, Sweden

**Keywords:** Client satisfaction, family planning, contraceptive use, multilevel analysis, health systems, quality of care, Ethiopia

## Abstract

**Background:**

Ethiopia has experienced growth in the utilization of family planning services. However, there are reports of relatively low client satisfaction across the country.

**Objective:**

The objective of this study was to assess client and facility level predictors of satisfaction with family planning services in Ethiopia.

**Method:**

A multi-level mixed effects logistic regression analysis was conducted on a national survey obtained from the service provision assessment 2021–22. A total of 2071 clients (level one) and 529 facilities (level two) were included.

**Results:**

Overall, 56% of clients were highly satisfied with the family planning service they received. Findings from the random effects of the multilevel analysis show there is a significant difference in client satisfaction between facilities, with an intra-class correlation of 0.56 in the null model. At the client-level, age above 30, auditory privacy, and discussion on side effects are significantly associated with higher client satisfaction. At the facility level, having a waiting area, facilities with a quality unit and/or committee, a DHIS2 reporting system, operating for more than 20 days a month, privately owned facilities and availability of family planning guidelines are associated with higher odds of being satisfied. On the other hand, at the client level, having a primary or higher education, increasing wait time and clients asking their providers questions are associated with lower odds of being satisfied. At the facility level, having a fixed user fee significantly reduces the odds of client satisfaction.

**Conclusions:**

Human resource and professional development training and health system strengthening is recommended.

## Paper context

- **Main findings:** Client satisfaction with family planning services in Ethiopia was found to be lower when compared to other low-income countries. It is influenced by both client-level and facility-level variables. Most of the modifiable factors that are associated with low client satisfaction are on the facility level.
- **Added knowledge:** A dedicated waiting area, establishing a quality unit, using the DHIS2 health information management system, operating for more than 20 days a month and maintaining auditory privacy were associated with higher client satisfaction. On the other hand, facilities charging a fixed user fee led to lower client satisfaction. In addition, a client with a higher educational background and one who asks questions is more likely to have lower client satisfaction.
- **Global health impact for policy and action:** Developing countries in a similar context can use the findings of this study to recognize areas of focus in regard to improving client satisfaction with family planning services.

## Background

According to the World Health Organization’s definition, family planning is a means of allowing people to attain their desired number of children and to determine the spacing of their pregnancies [1]. The first programs of modern family planning services began in the 1950s offering a limited range of methods [2]. However, these programs have been quickly recognized as key public health interventions for a mix of demographic, health, and human rights reasons [3].

The availability of modern contraceptives is a vital element in achieving sustainable global development as it contributes to poverty reduction and environmental sustainability [4]. It supports women’s empowerment and gender equality. It also promotes overall health, including lowering maternal morbidity and mortality and increasing life expectancy. Modern contraception helps in reducing the incidence of unsafe abortions and enhances child survival rates by allowing for appropriate birth spacing [4].

Most governments of developing countries have policies that favour lower rates of population growth and lower fertility [3]. However, contraceptive use varies significantly by region, ranging from 34 to 35% in Sub-Saharan Africa, Northern Africa and Western Asia to 50–60% in Latin America [5].

In Ethiopia pioneered by the Family Guidance Association, modern family planning services were introduced in 1966 [6]. Since then, the country has seen consistent growth in modern family planning utilization, the latest report reaching 41% among married women in 2019 [7]. A study analysing the trend in modern contraceptive use within the country reported an annual average increment of 1.9% from 2000 to 2016 [6]. Despite this growth, the total fertility rate (4.8) and unmet family planning need (25%) is still relatively high [8].

Disparities in family planning use, access, and issues surrounding suboptimal quality remain as challenges in Ethiopia. The quality of family planning services, measured by an average score of recommended clinical actions conducted during consultation, is reportedly low at the national level; recommended clinical steps were observed only one-third of the time [9]. This is reflected by the consistent reports of relatively low client satisfaction with family planning services ranging from 41 to 75% across several regions of Ethiopia [2,10–16]. The prevalence of client satisfaction with family planning services in other developing countries such as Tanzania (99%), Mozambique (86%), Mexico (80%) and Nepal (89%) is reportedly higher [10].

Various studies pointed out that client satisfaction with family planning services in Ethiopia is influenced by different factors. Socio-demographic factors such as education, residence and age have been shown to be associated with client satisfaction. Service-related factors such as wait time, privacy, comprehensive counselling including a discussion on how-to-use, side effects, concerns and follow up have been shown to be related with client satisfaction [2,10,14–16]. In addition, respectful and friendly approach by providers [14,15], anamnesis and physical examination [17] and asking partner’s attitude [18] also affect clients’ report of service satisfaction. Facility and administration factors such as opening hours, distance, ownership, family planning guideline availability [15,19] have also been reported to be associated with client satisfaction.

However, most studies assessing client satisfaction with family planning services in Ethiopia are done in a single facility setting; even when multiple facilities are sampled the analysis often does not take into consideration the hierarchy of the variables. The objective of this paper is to assess the client and facility level predictors of satisfaction with family planning services at a national level. The findings are important for designing strategic health policy and increasing the overall quality and responsiveness of family planning services in Ethiopia.

## Methods

### Data source and design

The Ethiopian Service Provision Assessment (ESPA) survey, carried out in 2021/22, was the second nation-wide evaluation of health facilities within Ethiopia. The ESPA 2021–22 survey encompassed an array of components, including facility inventory assessment, client exit interviews, observation of client–provider interactions, and interviews with health providers. The Ethiopian Public Health Institute (EPHI), in partnership with the Ministry of Health, executed the survey and technical support was provided by Inner City Fund (ICF) International.

This study employed a secondary analysis of data obtained from ESPA 2021/22 survey. Three data collection tools – namely, client-provider observation, client exit interview and facility inventory data were merged for this analysis.

Because of the non-proportional allocation of the sampled health facilities to the different regions, and the different health facility types, sampling weights are required for any analysis using the 2021–22 ESPA data. Therefore, before analysis, client weights for descriptive statistics and facility weights for multilevel models were used to restore the actual representativeness of the sample to determine the proportion of distribution of client satisfaction across different explanatory variables.

An in-depth explanation of the ESPA instruments, methodologies and procedures are described elsewhere [7].

## Setting

The study was conducted in Ethiopia across its nine regions and two city administrations. As of 2023, Ethiopia boasts a population of 128 million, positioning it as the second most populous country in Africa and it is projected to reach 147 million in 2030 [20]. The country is characterized by rapid population growth at a rate of 2.6%, a young age structure, and a high dependency ratio [7]. According to the World Bank, in 2021, the total fertility rate in Ethiopia was 4.6 births per woman (2.3 in urban areas and 5.2 in rural areas) and the crude birth rate was 32 per 1,000 [21].

## Participants/study size

The survey selected a stratified random sample of 1,407 health facilities, selected with equal probability systematic sampling. However, data was successfully collected from 1158 facilities; the remaining were permanently closed, not yet operational, under security issues, converted into a COVID Centre, unreachable, or duplicates of another facility in the sample. Among these facilities, 1046 are reported to ‘offer’ family planning services, which includes facilities that counsel and prescribe family planning methods for a client to obtain elsewhere. Of these facilities, 648 provide family planning services within the facility.

**Figure 1.**
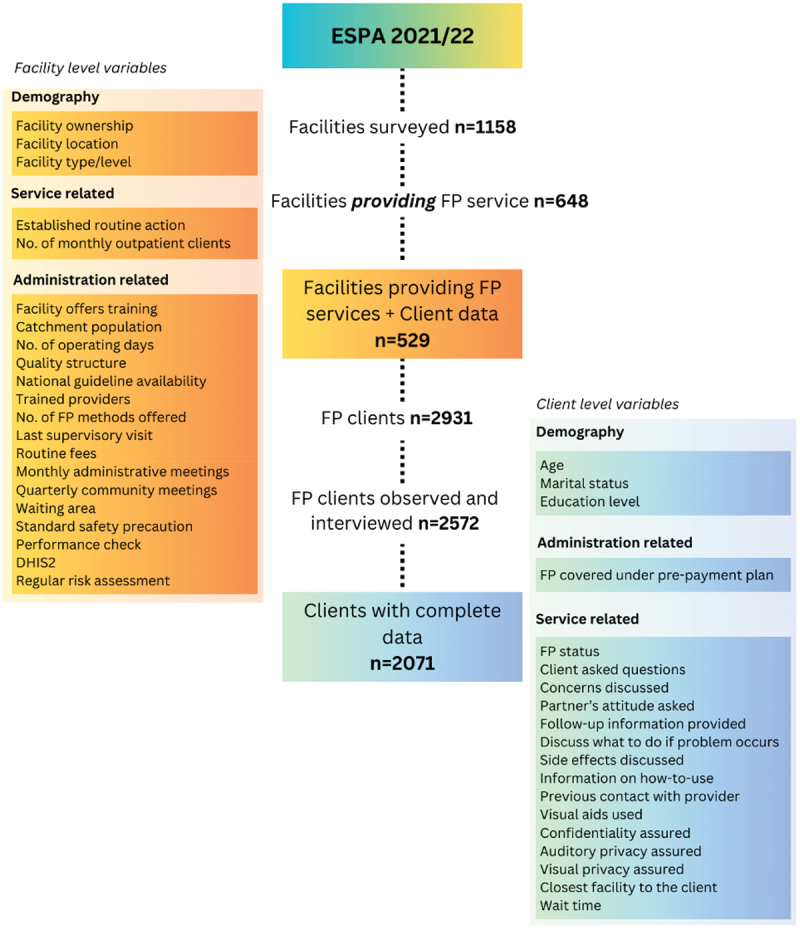
Flowchart of the sampling procedure and the variables included under each level.

The number of eligible clients, nested under the sample of facilities, present on the day of the survey for family planning services was 2,931; among which 2572 were both observed and interviewed in a subset of 529 facilities. From our analysis, 501 client samples were excluded due to incompleteness, including refusal to participate in interview. The final analysis was based on 2071 clients nested in 529 facilities. (See Figure 1)

## Variables

### Outcome variable

The outcome variable for this study was client satisfaction.

Client satisfaction for this study was measured from the client exit interview question about the overall satisfaction of clients with the family planning service received at the facility. The Likert scale for the question included four levels: namely, very satisfied, somewhat satisfied, somewhat dissatisfied, and very dissatisfied. The response was dichotomized by grouping the first level into ‘very satisfied’ and the last three levels into ‘not very satisfied’. Thereafter, these groups were named ‘more satisfied’ and ‘less satisfied’, respectively. This nomenclature reflects the continuum of client satisfaction, where the less satisfied group logically includes varying degrees of dissatisfaction. By framing satisfaction in this manner, the analysis maintains rigor, and simplifies understanding for readers, while aligning with the study’s goal of identifying predictors to maximize the ‘very satisfied’ category.

### Independent variables

A total of 40 explanatory variables were included in the analysis. Nineteen [19] of which were from client level and 21 from the facility level. The variables included demographic, administration-related and service-related characteristics (See Figure 1). A detailed description of each variable is provided in the Appendix (Table A1).

## Statistical methods

All analyses were performed using R Statistical Software (v4.3.1; R Core Team 2023) [22]. Summary statistics were conducted to compare the frequency of characteristics between ‘more satisfied’ and ‘less satisfied’ categories on both client and facility levels. These analyses were conducted after applying client weights to adjust for the complex and disproportionate sampling technique by using the ‘survey’ R package (v4.4.2; T. Lumley 2024) [23] (See Table 1). Multilevel mixed effects analysis was done using the ‘lme4’ R package (v1.1.34; Bates et al 2015) [24].

**Table 1.** Frequency of socio-demographic variables with and without weights.

Client level variables	Category	n (%) unweighted	n (%) weighted
Age	<22	533 (25.7)	509 (24.6)
	22–30	1159 (56.0)	1121 (54.1)
	>30	379 (18.3)	441 (21.3)
Marital status	Not married	160 (7.7)	131 (6.3)
	Married	1911 (92.3)	1940 (93.7)
Educational status	No education	503 (24.3)	637 (30.8)
	Primary education	718 (34.7)	841 (40.6)
	Higher education	850 (41.0)	593 (28.6)
Facility level variables			
Ownership	Public	1914 (92.4)	1881(90.8)
	Private	157 (7.6)	190 (9.2)
Location	Urban	1305 (63.0)	858 (41.4)
	Rural	766 (37.0)	1213 (58.6)
Level	Hospital	1189 (57.4)	162 (7.8)
	Health centre	707 (34.1)	1169 (56.5)
	Clinic	108 (5.2)	556 (8.9)
	Health post	67 (3.2)	184 (26.8)

Multilevel mixed effects logistic regression analysis was used to examine the magnitude of association and measure of variation between two level independent variables and client satisfaction. The reason to perform a multilevel regression analysis was mainly because the collected data has hierarchical structure where clients (level one) were nested under facilities (level two) which results in client satisfaction being correlated among clients receiving family planning service under the same facility. This breaks the rule of independence and equal variance to perform a standard logistic regression.

Even though it is possible to perform a single level logistic regression using only client level or only facility level variables, this approach will highly restrict the explanation for the outcome variable. Client satisfaction cannot be comprehensively explained by considering only client or facility level variables. The analysis needs to account for the interaction between and within these two levels to estimate association with the outcome variable more accurately and avoid biased results. A multilevel model allows us to consider both the client and the facility level in the same analysis [19].

Four models containing variables of interest were fitted. Null model (empty model) contained no independent variables and only focused on decomposing the total variance into its client and facility components; meaning no fixed effects were calculated. Model I contained only client-level variables and Model II included only facility-level variables. Model III contained variables with a p-value of less than 0.2 from both client and facility levels. The p-value cut off was selected in accordance with research utilizing a similar methodology [19].

The variable ‘region’ has been tested as third level in the multilevel analysis. However, it was statistically insignificant in attributing the variation observed in client satisfaction (*ICC* 0.08 *p-value* 0.093).

## Random effects (measures of variation)

Random effects or measures of variation such as Intra-class Correlation Coefficient (ICC), Proportional Change in Variance (PCV) and Median Odds Ratio (MOR) were computed to measure the heterogeneity of client satisfaction across facilities. Considering facilities as a random variable, the Intra-class Correlation Coefficient quantifies the degree of variation of client satisfaction between facilities (the proportion of the total observed variation in client satisfaction that is attributable to facility level variance (Vf)) is computed as; ICC= (Vf/(Vf + 3.29)) * 100. The Median Odds Ratio (MOR) represents the median value of the odds ratio, serving as a quantifier of the heterogeneity in client satisfaction across different facilities on an odds ratio scale. It is characterized as the median value of the odds ratio between a facility with superior client satisfaction and a facility with inferior client satisfaction when clients are randomly selected from two facilities [25]; MOR= exp (0.95√Vf).

Moreover, the PCV demonstrates the variation in client satisfaction explained by the variables in the model and it is computed as; PCV= (Vnull – Vf/Vnull)*100; where Vnull = variance of the null model and Vf = facility level variance [26].

## Fixed effects (measures of association)

The association between client satisfaction and independent variables at both the client and facility levels was estimated using fixed effects. This association was evaluated, and its magnitude was expressed using the Adjusted Odds Ratio (AOR) along with 95% confidence intervals, considering a p-value of less than 0.05 as statistically significant.

Given that this study employed multiple explanatory variables that could potentially be correlated, a test for multicollinearity was conducted using the means of the Variance Inflation Factors (VIFs). Multicollinearity was defined as tolerance values below 0.1 and VIF values equal to or greater than 10. Except for the facility region all variables showed a small VIF of maximum 2.56 indicating the absence of any significant collinearity between explanatory variables. The facility region was excluded from the regression model.

Log likelihood ratio (LLR) was used to compare models, due to the nested nature of the data, and the model with the highest LLR was selected as the best-fit model. Akaike Information Criterion (AIC) and Bayesian Information Criterion (BIC) are also reported.

## Results

### Sociodemographic characteristics

A total of 2071 clients (level one) and 529 facilities (level two) were included in this study. The effect of adding weight is illustrated in Table 1 where both unweighted and weighted frequency tables are presented. Henceforth, for description and analysis weighted data was used to reflect the existing distribution pattern of client and facility demographic variables.

## Client satisfaction and sociodemographic variables

Out of the total participants, 56% (1161 clients) expressed higher satisfaction with family planning services, while the remaining 44% (910 individuals) reported lower satisfaction levels. Notably, a comparable distribution of socio-demographic variables was observed across both the less satisfied and more satisfied groups, at both the client and facility levels (See Table 2).

**Table 2.** Frequency of socio-demographic variables by client satisfaction (column percentages are reported).

Variables	Category	Less Satisfied, N = 910	More Satisfied, N = 1,161
Client level variables			
Age	Below 22	262 (28.8%)	247 (21.3%)
	22–30	523 (57.4%)	599 (51.5%)
	>30	125 (13.8%)	315 (27.2%)
Marital status	Not married	56 (6.2%)	75 (6.5%)
	Married	854 (93.8%)	1,086 (93.5%)
Education level	No Education	240 (26.3%)	397 (34.3%)
	Primary	409 (45.0%)	432 (37.2%)
	Higher	261 (28.7%)	332 (28.5%)
**Facility level variables**			
Ownership	Public	854 (93.9%)	1,026 (88.4%)
	Private	56 (6.1%)	135 (11.6%)
Location	Urban	328 (36.0%)	530 (45.7%)
	Rural	582 (64.0%)	631 (54.3%)
Level	Hospital	67 (7.4%)	95 (8.2%)
	Health Centre	491 (53.9%)	678 (58.4%)
	Health Post	298 (32.8%)	258 (22.2%)
	Clinic	54 (5.9%)	130 (11.2%)

## Client satisfaction and service-related variables

In Table 3, among the clients expressing less satisfaction with family planning services, 84.1% were current users of these services. 59.2% refrained from asking questions to their service providers, and 51.5% did not voice their concerns. 95% were not asked about their partner’s perspective on their chosen family planning method. In 88.8% of the consultations observed, visual aids were not utilized to demonstrate the methods. Confidentiality was not guaranteed in 81.2% of the observed cases, and 52.5% of the consultations took place in a facility lacking established routine procedures prior to the provision of family planning services.

**Table 3.** Frequency of service-related variables by client satisfaction (column percentages are reported).

Variables	Category	Less Satisfied, N = 910	More Satisfied, N = 1,161
Family planning status	Current User	766 (84.1%)	892 (76.9%)
	Past User	77 (8.5%)	170 (14.6%)
	Never Used	67 (7.4%)	99 (8.5%)
Client asked questions	No	538 (59.2%)	800 (68.9%)
	Yes	372 (40.8%)	361 (31.1%)
Concerns discussed	No	468 (51.5%)	706 (60.9%)
	Yes	442 (48.5%)	454 (39.1%)
Partner’s attitude asked	No	864 (95.0%)	1,080 (93.0%)
	Yes	46 (5.0%)	81 (7.0%)
Follow-up information	No	30 (3.3%)	31 (2.6%)
	Yes	880 (96.7%)	1,130 (97.4%)
Discussion if problem occurs	No	460 (50.6%)	403 (34.7%)
	Yes	450 (49.4%)	758 (65.3%)
Side effects discussed	No	434 (47.7%)	411 (35.4%)
	Yes	476 (52.3%)	750 (64.6%)
Information on how-to-use	No	235 (25.9%)	250 (21.5%)
	Yes	675 (74.1%)	911 (78.5%)
Previous contact	No	205 (22.5%)	260 (22.4%)
	Yes	705 (77.5%)	901 (77.6%)
Visual aids used	No	808 (88.8%)	964 (83.0%)
	Yes	102 (11.2%)	197 (17.0%)
Confidentiality assured	No	739 (81.2%)	911 (78.5%)
	Yes	171 (18.8%)	250 (21.5%)
Auditory privacy assured	No	259 (28.4%)	267 (23.0%)
	Yes	651 (71.6%)	894 (77.0%)
Visual privacy assured	No	180 (19.8%)	211 (18.1%)
	Yes	730 (80.2%)	950 (81.9%)
Nearest facility for client	No	78 (8.5%)	112 (9.6%)
	Yes	832 (91.5%)	1,049 (90.4%)
Routine action established	No	478 (52.5%)	648 (55.8%)
	Yes	432 (47.5%)	513 (44.2%)

On the other hand, among clients who reported to be more satisfied with the family planning service only 31.1% asked questions to their providers and 39.1% discussed their concerns. 7% of the clients who reported higher satisfaction were asked about their partner’s attitude, 17% of the consultations utilized visual aids and 21.5% of the time was confidentiality assured.

## Client satisfaction and administration related variables

In Table 4, among clients who were less satisfied with the family planning service 64% were provided in a facility that did not offer training to providers and 65.8% were in a facility that did not have at least one provider who was trained within the last two years. From clients who reported less satisfaction 57.8% were in facilities that serve a population of less than 25,000. 70.2% of clients who reported less satisfaction were from facilities that operated for fewer than 20 days per month. The absence of a quality structure also contributed to dissatisfaction, with 46.9% of clients with less satisfaction being from facilities without such a quality structure.

**Table 4.** Frequency of administration-related variables by client satisfaction (column percentages are reported) *.

Variables	Category	Less Satisfied, *N* = 910	More Satisfied, *N* = 1,161
Facility offers training on FP	No	583 (64.0%)	747 (64.4%)
	Yes	327 (36.0%)	414 (35.6%)
Catchment population	Below 25K	526 (57.8%)	595 (51.3%)
	25K to 1.5M	329 (36.2%)	421 (36.3%)
	>1.5M	55 (6.0%)	145 (12.4%)
No. of days facility operates	Below 20	638 (70.2%)	799 (68.8%)
	Above 20	272 (29.8%)	362 (31.2%)
Quality structure	No Structure	426 (46.9%)	345 (29.8%)
	Quality Committee	336 (36.9%)	596 (51.2%)
	Unit & Committee	148 (16.2%)	220 (19.0%)
National FP guideline	No	394 (43.3%)	393 (33.9%)
	Yes	516 (56.7%)	768 (66.1%)
Trained providers	No	598 (65.8%)	822 (70.9%)
	Yes	312 (34.2%)	339 (29.1%)
No. of FP methods offered	<8	495 (54.4%)	647 (55.8%)
	≥8	415 (45.6%)	514 (44.2%)
Prepayment plan	No	617 (67.8%)	777 (66.9%)
	Yes	293 (32.2%)	384 (33.1%)
Last supervisory visit	Never	656 (72.1%)	836 (72.0%)
	Within 6 months	93 (10.2%)	118 (10.1%)
	>6 months	161 (17.7%)	207 (17.9%)
Routine fees	None	284 (31.2%)	240 (20.7%)
	Fixed	140 (15.4%)	80 (6.9%)
	Separate	486 (53.4%)	841 (72.4%)
Monthly administrative meetings	No	135 (14.9%)	201 (17.3%)
	Yes	775 (85.1%)	960 (82.7%)
Quarterly community meetings	No	336 (36.9%)	500 (43.1%)
	Yes	574 (63.1%)	661 (56.9%)
Dedicated waiting area	No	178 (19.6%)	122 (10.5%)
	Yes	732 (80.4%)	1,039 (89.5%)
Standard safety precautions	No	590 (64.9%)	699 (60.2%)
	Yes	320 (35.1%)	462 (39.8%)
Facility performance check	None	310 (34.1%)	339 (29.2%)
	Clinical audit	205 (22.5%)	265 (22.9%)
	Regular audit	324 (35.6%)	387 (33.2%)
	Feedback	71 (7.8%)	170 (14.7%)
DHIS2 reporting system available	No	499 (54.8%)	526 (45.3%)
	Yes	411 (45.2%)	635 (54.7%)
Regular risk assessment conducted	No	665 (73.0%)	856 (73.8%)
	Yes	245 (27.0%)	305 (26.2%)

*Reported counts in the table represent the **number of clients** who received services in facilities with the specified characteristics.

Furthermore, 54.4% of clients with less satisfaction were from facilities that offered fewer than 8 family planning methods. A higher proportion (67.8%) of less satisfied clients were from facilities that did not include family planning services as part of a prepayment plan. 72.1% of less satisfied clients were from facilities that have never had a supervisory visit.

Facilities that charged a separate routine fee for family planning services reported a higher proportion (53.4%) of less satisfied clients. Non-adherence to standard safety precautions resulted in 64.9% of less satisfied clients being from these respective facilities. Facilities that did not conduct regular risk assessments reported a higher proportion (73%) of less satisfied clients. In addition, facilities that did not use the District Health Information Software (DHIS2) also reported a higher proportion (54.8%) of less satisfied client. These factors collectively contributed to the overall dissatisfaction reported in these facilities.

On the other hand, clients still reported being less satisfied in higher proportions despite the facility has availed family planning guidelines (56.7%), conducted monthly administrative meetings (85.1%) and quarterly community meetings (63.1%), had a dedicated waiting area (80.4%) and regular audit (35.6%).

## Random effect (measures of variation) and model fit

In Table 5, findings from the null model showed that there are significant differences in client satisfaction between facilities, with a variance of 4.2. In the null model, about 56% of the total variation in client satisfaction was attributable to the facility-level factors. In addition, the highest median odds ratio (MOR) value (7.0) was seen in the null model. This means that, when choosing a client at random from one facility at a lower client satisfaction and another facility at a higher client satisfaction, those in the lower satisfaction facility have 7 times higher odds of being less satisfied than those in the higher satisfaction facility.

**Table 5.** Random effects parameters and model fit comparison.

Parameter	Null model	Model I	Model II	Model III
Facility level variance	4.20	3.40	2.72	2.10
ICC	0.56	0.51	0.45	0.39
MOR	7.00	5.76	4.78	3.96
PCV	Reference	19.09%	35.33%	50.04%
Model fit				
LLR	−963.52	−904.52	−902.78	−846.16
AIC	1931.06	1859.04	1861.56	1756.32
BIC	1939.60	1965.81	1981.15	1893.00

The intraclass correlation (ICC) value for the final model (model III) was 39% indicating that more than one-third of the variation in client satisfaction is attributed to unobserved facility-level factors. Additionally, 50.04% of the variance in the odds of having less or more client satisfaction is explained by both client- and facility-level factors in the final model, as indicated by the proportional change in variance (PCV).

The model fit comparison was conducted using Log Likelihood Ratio (LLR). Akaike Information Criterion (AIC) and Bayesian Information Criterion (BIC) are also reported. The final model (model III) was the best-fitted model since it had the highest log likelihood ratio (−846.16) and lowest AIC and BIC which were 1756.32 and 1893.00, respectively.

## Multivariable multilevel logistic regression analysis

In Table 6 the three models are presented, model I with only client-level variables, model II with only facility-level variables and the final model, model III which consists of variables from both the client and facility levels.

**Table 6.** Multivariable multilevel mixed-effect logistic regression.

Variables	Category	Model I Model II Model III * AOR (95% CI) AOR (95% CI) AOR (95% CI)		
Client level variables				
Age	<22 1 1			
	22–30	**1.48 (1.02–2.14)**		1.25 (0.87–1.81)
>30		**3.33 (2.02–5.49)**		**2.99 (1.82–4.91)**
Educational level	No education	1		1
	Primary	**0.57 (0.39–0.84)**		**0.54 (0.38–0.79)**
	Higher	0.74 (0.48–1.14)		**0.63 (0.41–0.97)**
Waiting time		**0.99 (0.99–1.00)**		**0.99 (0.98–1.00)**
Auditory privacy	No	1		1
	Yes	**1.72 (1.01–2.95)**		**1.73 (1.17–2.55)**
Family planning status	Current user	1		1
	Past user	**1.73 (1.05–2.83)**		1.56 (0.97–2.51)
	Never used	1.63 (0.85–3.10)		1.45 (0.83–2.53)
Client asked questions	No	1		1
	Yes	**0.52 (0.34–0.79)**		**0.47 (0.33–0.67)**
Discussion on problems	No	1		1
Yes 2.97 (1.83–4.84) 2.96 (2.10–4.19)				
Facility level variables				
Waiting area No 1 1				
	Yes		**7.25 (3.50–15.05)**	**5.62 (2.96–10.66)**
Number of FP methods	<8		1	1
	≥8		**0.55 (0.31–0.96)**	0.75 (0.45–1.26)
Quality structure	None		1	1
	Quality unit alone		**5.07 (2.56–10.04)**	**3.57 (1.93–6.59)**
	Quality unit & committee		**4.78 (1.83–12.47)**	**3.60 (1.54–8.54)**
Routine fee	None		1	1
	Fixed		**0.06 (0.02–0.19)**	**0.07 (0.03–0.20)**
	Separate		**0.46 (0.22–0.97)**	0.63 (0.31–1.25)
DHIS2	No		1	1
	Yes		**2.54 (1.33–4.84)**	**2.30 (1.27–4.16)**
Opening days per month	<20		1	1
	≥20		1.67 (0.97–2.88)	**1.70(1.02–2.84)**
Ownership	Public		1	1
	Private		2.25(0.71–7.17)	**3.28(1.29–8.33)**
National guideline	No		1	1
	Yes		1.40 (0.85–2.30)	**1.64(1.05–2.56)**

*In addition to the variables listed in the table history and physical examination, visual aids, counselling on how to use, facility offers training, monthly meeting, supervisory visit, and performance check are also controlled for in model III.

In the multivariable multilevel mixed-effect logistic regression analysis, where both the client and facility-level factors were fitted simultaneously, age, educational level, wait time, auditory privacy, clients asking questions, discussion on what to do if a problem occurs, waiting area, quality structure, routine fee, the presence of DHIS2 reporting system, the number of days facility operates, facility ownership and availability of national guideline on family planning were significantly associated with client satisfaction at a p-value of < 0.05.

Results from the final model (model III) reported as (AOR, (95%CI)) revealed that clients with primary and secondary education (0.54, (0.38–0.79); 0.63, (0.41–0.97), respectively) had lower odds of being satisfied with family planning services when compared to those with no education. A one percent decline in odds of being satisfied is observed for each minute increase in wait time (0.99, (0.98–1.00)). Clients who asked questions to their providers regarding their method of family planning choice (0.47, (0.33–0.67)) had lower odds of being satisfied. A facility with a fixed routine fee for family planning services (0.07, (0.03–0.20)) experienced lower client satisfaction when compared to a facility with no fees.

Conversely, clients aged more than 30 (2.99, (1.82–4.91)) had higher odds of being satisfied with family planning service when compared to clients aged below 22. Maintaining auditory privacy (1.73, (1.17–2.55)) and having discussions on what to do if a problem occurs (2.96, (2.10–4.19)) lead to higher odds of being satisfied. A facility with a dedicated waiting area (5.62, (2.96–10.66)) increased the odds of a client being satisfied by 5-fold. Similarly, facilities with a quality unit alone or both quality unit and quality committee (3.57, (1.93–6.59); 3.60, (1.54–8.54) respectively) increased the odds of client satisfaction. Facilities using DHIS2 reporting system (2.30, (1.27–4.16)) and operating for more than 20 days a month (1.70, (1.02–2.84)) showed a significant association with higher client satisfaction. In addition, the type of facility played a role in satisfaction levels. Clients receiving services in private facilities (3.28, (1.29–8.33)), as opposed to public ones, reported significantly higher satisfaction. Lastly, the availability of national guidelines at the facility was associated with increased client satisfaction (1.64, (1.05–2.56)).

## Discussion

The objective of this study was to assess the client and facility-level predictors of satisfaction with family planning services at a national level.

The findings of this study showed that 56% of clients were ‘more satisfied’ with family planning services in Ethiopia. This is consistent with the family planning service satisfaction rate (41 to 75%) that is reported across the country [2,10–16]. However, it is much lower than other low-income countries such as Kenya (93%), Tanzania (99%), Mozambique (86%) and Nepal (89%) [10,27].

In Table 6, the findings demonstrate that both client-level and facility-level factors were associated with family planning service satisfaction in Ethiopia. At the client-level, age, educational level, wait time, auditory privacy, client asking questions to their provider and discussion on what to do if a problem occurs were significantly associated with client satisfaction. At the facility-level, having a dedicated waiting area, quality structure, presence of routine user fee, DHIS2 reporting system, number of operating days, facility ownership and the availability of national guidelines were significantly associated with client satisfaction.

A dedicated waiting area for family planning services is significantly associated with higher client satisfaction. According to the availability, accessibility, acceptability, and quality (AAAQ) framework, this is an expected finding because a health facility that is designed taking the comfort of patients into consideration is reflective of the attention given to acceptability and quality of a service [28]. In line with this, facilities which have a quality unit, or a quality committee are also associated with higher client satisfaction. Similarly, having an electronic medical record such as DHIS2 can be seen in the same light. Electronic medical records have been shown to increase the quality of health service delivery including timely and patient-centred care [29,30]. Moreover, a systematic review on the use of electronic medical records in the examination room reported positive effects on patient satisfaction [31].

From this study, receiving a family planning service in a private facility resulted in higher client satisfaction. Similar reports have been made in a study analysing findings from seven African countries; namely Egypt, Kenya, Senegal, Ethiopia, Ghana, Tanzania, and Namibia where the authors concluded quality family planning services were ‘positively associated with privately owned facilities’ [32]. In addition, a study in Kenya showed client satisfaction with family planning services being higher in private facilities because of process and structural factors [33]. However, another study conducted in Ethiopia from a national survey reported that women who received their family planning service from a private facility had worse counselling than public facilities [9]. Further research is required to determine the factors associated with facility ownership and quality of family planning services.

In this study, adult women more than the age of 30 were more likely to be satisfied with the family planning service they received (2.99 (1.82–4.91)) when compared to women aged less than 22. This finding is similar to studies conducted in Southern Ethiopia [15] and Northern Ethiopia [14] where older clients were found to be more satisfied with their family planning services than the younger clients. It is also consistent with a report from analysis of a national survey by Hrusa et al where they found women who were more than the age of 25 more likely to receive a better-quality family planning counselling when compared to adolescents [9]. A possible explanation for this is the numerous challenges and barriers youth face in accessing sexual and reproductive health (SRH) services. A study in different countries within Africa mentions personal biases and unfriendly attitudes of providers towards adolescents and a working environment that is poorly equipped to deal with young people as major barriers to access quality family planning services for young people [34].

In this study, discussion on what to do if a problem occurs during contraception usage has been found to be a significant factor in making clients more satisfied with the service. This finding is in line with other studies in Ethiopia [14–16]. In addition, Tessema et al also reported higher satisfaction among clients who received adequate information on potential side effects of the chosen contraceptive [19].

Clients whose auditory privacy was maintained were more satisfied with the family planning service they received in this study. This is similar to the studies done in Southern Ethiopia [15,16], Eastern Ethiopia [35] and several countries in Africa [32]. Despite repeated evidence for the importance privacy plays in assuring client satisfaction, almost a quarter of the clients in this study did not have their auditory privacy maintained.

In line with the importance of availability and accessibility, facilities that operate for more than 20 days a month are associated with higher client satisfaction in this study. This finding is similar to studies done in Southern and Eastern Ethiopia where convenient opening hours are reported to be significantly associated with client satisfaction [15,35].

The availability of family planning guidelines for providers in a facility is a significant determining factor for client satisfaction. Tessema et al. also reported a similar finding from their analysis of the 2014 Ethiopian service provision assessment dataset [19]. This is in line with existing literature because the availability of guidelines in facilities allow healthcare providers the opportunity to stay up to date with current medical knowledge and practice, which in turn leads to better quality counselling.

Waiting time has been consistently shown to be a determining factor resulting in less satisfaction with family planning services. That is also the case in this study and several other studies in Ethiopia and Sub-Saharan Africa [16,36,37]. Tessema et al also found, using the 2014 Ethiopian service provision assessment dataset, longer waiting times to be a significant determinant of client dissatisfaction [19].

This study found that clients with a primary and secondary education were less satisfied with the family planning service they received when compared to clients with no educational background. This is different from a study done in Sub-Saharan Africa that reported women with higher educational level being more satisfied with their family planning service [5]. However, a study in the southern part of Ethiopia found women with a college education and above were less satisfied with the family planning service [16]. A possible explanation for this can be the varying expectations between women with and without education and the degree to which these expectations are met by the provider and the facility. Reports show that, in some situations health care providers often lack adequate knowledge and skills with regard to contraceptive services [34]. A qualitative study from Ethiopia also stated that women are sometimes misinformed and pressured in to using a certain contraceptive method regardless of their preference [38]. These factors tend to be easily noticed by educated clients, causing them to be less satisfied with the service.

In this study, clients who asked their providers questions were less likely to be satisfied with the service they received. This could be related to the socio-demography of clients who are more likely to ask questions and the capability of their providers to answer those questions. The discrepancy between an inquisitive client and a less capable provider could lead to patient dissatisfaction. However, further research in this area is warranted.

Facilities with fixed routine user fees for family planning services are associated with significantly less client satisfaction. This finding aligns with a study conducted in 36 low- and middle-income countries which reported user fees and out of pocket expenses as barriers to access quality sexual and reproductive health services [39].

## Limitations

Factors associated with healthcare providers can influence client satisfaction. Nonetheless, in the 2021–22 ESPA the random sample of providers is not linked to the clients they have consulted and/or treated. Therefore, provider-level variables could not be included in the analysis. Another limitation is that data collection through observation of client–provider interaction might produce the ‘Hawthorne effect’ wherein providers alter their usual behavior to appear more favorable, potentially inflating satisfaction levels. In addition, social desirability bias during client exit interviews may lead respondents to overreport satisfaction, further skewing the results toward higher satisfaction levels.

## Generalizability

This study analysed a nationally representative dataset and implemented a multi-level methodology to address the hierarchy of the numerous explanatory variables. The findings have expanded the existing literature and can be used as a baseline for further study in this area. It can also be used to assess the status of client satisfaction and identify areas of improvement in family planning services in Ethiopia.

## Conclusion

**Figure 2.**
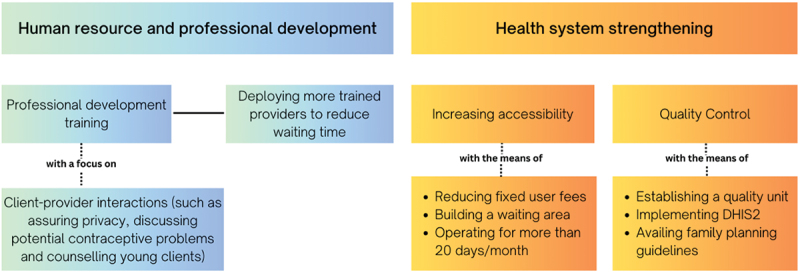
Policy recommendations to increase client satisfaction with family planning services.

The findings of this study indicate that the growth in the provision of modern contraception in Ethiopia has not been accompanied by a due attention to client satisfaction, evidenced by the relatively low proportion of highly satisfied clients when compared to other low-income countries. Family planning service satisfaction is influenced by both client-level and facility-level variables. Most of the modifiable factors that are associated with low client satisfaction are on the facility level. Based on this study, a policy recommendation figure has been produced, highlighting factors that are significantly associated with client satisfaction (See Figure 2).

## Supplementary Material

Supplementary material optional.docx

## Data Availability

The dataset used for this study can be accessed via the demographic and health surveys (DHS) program.

## Acronyms

AOR: Adjusted Odds Ratio
DHIS2: District Health Information Software, Version 2
ESPA: Ethiopian Service Provision Assessment
FP: Family Planning
ICC: Intra-class Correlation Coefficient
LLR: Log Likelihood Ratio
MOR: Median Odds Ratio
PCV: Proportional Change in Variance

## Appendices Appendix 1

**Table A1.** List of dependent and independent variables by type and source dataset.

Variables	Type of variable	Source
Outcome variable		
Client satisfaction	Binary (‘more satisfied’ and ‘less satisfied’). Clients who reported being very satisfied are labeled as ‘more satisfied’ and those who reported being somewhat satisfied, somewhat dissatisfied, and very dissatisfied as labeled as ‘less satisfied’	Client exit interview
**Independent variables**		
**Client level variables**		
Age	Numeric	Client exit interview
Marital status	Categorical (‘not married’ and ‘married’); those who are single, widowed, divorced, and separated were labeled as ‘not married’	Client exit interview
Educational level	Categorical (‘No education’, ‘Primary education’ and ‘Higher education’); secondary education is also labeled as higher education	Client exit interview
Information provided about how to use the contraceptive method	Binary (‘yes’ and ‘no’); records of ‘don’t know’ were labeled as ‘no’	Client-provider observation
Information provided about the side effects of the chosen contraceptive method	Binary (‘yes’ and ‘no’); records of ‘don’t know’ were labeled as ‘no’	Client-provider observation
Information provided on what to do if a problem occurs	Binary (‘yes’ and ‘no’); records of ‘don’t know’ were labeled as ‘no’	Client-provider observation
Information provided on when to return for follow up	Binary (‘yes’ and ‘no’); records of ‘don’t know’ were labeled as ‘no’	Client-provider observation
Partner’s attitude towards the use of the chosen contraception is asked	Binary (‘yes’ and ‘no’)	Client-provider observation
Concerns about the chosen contraception is asked	Binary (‘yes’ and ‘no’)	Client-provider observation
The client has asked questions regarding contraception methods	Binary (‘yes’ and ‘no’)	Client-provider observation
Client’s waiting time before seeing a provider	Numeric (in minutes)	Client-provider observation
Status of client’s contraception use	Categorical (‘Current user’, ‘Past user’ and ‘Never used’)	Client exit interview
Family planning service covered under a pre-payment plan	Binary (‘yes’ and ‘no’); records of ‘don’t know’ were labeled as ‘no’	Client exit interview
Facility providing family planning is the closest to the client	Binary (‘yes’ and ‘no’); records of ‘don’t know’ were labeled as ‘yes’	Client exit interview
Visual privacy was assured during consultation and treatment	Binary (‘yes’ and ‘no’)	Client-provider observation
Auditory privacy was assured during consultation and treatment	Binary (‘yes’ and ‘no’)	Client-provider observation
Confidentiality was assured during consultation and treatment	Binary (‘yes’ and ‘no’)	Client-provider observation
Visual aids were used during consultation and treatment	Binary (‘yes’ and ‘no’)	Client-provider observation
The client had previous contact with the provider	Binary (‘yes’ and ‘no’); records of ‘don’t know’ were labeled as ‘no’	Client exit interview
**Facility level variables**		
Facility ownership	Categorical (‘public’ and ‘private’)	*Facility inventory*
Facility location	Categorical (‘urban’ and ‘rural’)	*Facility inventory*
Facility type	Categorical (‘hospital’, ‘clinic’, ‘health center’ and ‘health post’); all levels of hospitals and clinics were aggregated together	*Facility inventory*
Facility has an established routine action	Binary (‘yes’ and ‘no’)	*Facility inventory*
Facility has national guidelines on family planning readily available	Binary (‘yes’ and ‘no’)	*Facility inventory*
Facility has an established quality assurance structure	Categorical (‘No structure’, ‘Only quality committee’ and ‘Both quality unit and quality committee’)	*Facility inventory*
Number of days the facility offers family planning services	Categorical (‘Below/equal to 20 days’ and ‘Above 20 days’)	*Facility inventory*
Catchment population for the facility	Categorical (‘Below 25,000’, ‘25,000 to 1,500,000’ and ‘Above 1,500,000’)	*Facility inventory*
Facility offers training on family planning services	Binary (‘yes’ and ‘no’)	*Facility inventory*
Facility has providers who took training on family planning within the last two years	Binary (‘yes’ and ‘no’)	*Facility inventory*
When was the last time the facility had a supervisory visit	Categorical (‘Never’, ‘Within the last 6 months’ and ‘More than 6 months ago’)	*Facility inventory*
What is the structure of routine fees for family planning services at the facility	Categorical (‘Fixed’, ‘Separate’ and ‘No routine fees’)	*Facility inventory*
Does the facility hold administrative meetings monthly	Binary (‘yes’ and ‘no’)	*Facility inventory*
Does the facility hold community meetings at least quarterly	Binary (‘yes’ and ‘no’)	*Facility inventory*
Number of patients seen on outpatient basis monthly	Numeric	*Facility inventory*
Facility has a waiting area for family planning clients	Binary (‘yes’ and ‘no’)	*Facility inventory*
Facility adheres to standard safety precautions for providing family planning services	Binary (‘yes’ and ‘no’)	*Facility inventory*
The type of performance checks the facility conducts	Categorical (‘No performance check’, ‘Regular audit’, ‘Clinical audit’ and ‘Feedback collection’)	*Facility inventory*
Facility uses DHIS2	Binary (‘yes’ and ‘no’)	*Facility inventory*
Facility conducts regular risk assessments	Binary (‘yes’ and ‘no’); records of ‘don’t know’ were labeled as ‘no’	*Facility inventory*
Number of family planning methods offered	Categorical (‘Less than 8’ and ‘More than or equal to 8’)	*Facility inventory*
