# Supplementary material for: A multilevel analysis on the predictors of client satisfaction with family planning services in Ethiopia: evidence from the Ethiopian service provision assessment (ESPA) 2021/22
Source: Glob Health Action. 2025 Feb 13;18(1):2463215. doi: 10.1080/16549716.2025.2463215 (PMC11827030; doi:10.1080/16549716.2025.2463215)
Supplement: Supplementary material optional.docx [file ZGHA_A_2463215_SM4291.docx]

Multilevel Modelling - Predictors of Client Satisfaction with Family Planning Services in Ethiopia

Michael Endale Mengesha and Henrik Holmberg

Department of Epidemiology and Global Health, Umeå University

Table of Contents

[Background 1](#_Toc184684543)

[Composite measure for client satisfaction 2](#_Toc184684544)

[Single variable measure for client satisfaction 3](#_Toc184684545)

[Weights 4](#_Toc184684546)

[Multilevel modelling 4](#_Toc184684547)

[Facility as second level 4](#_Toc184684548)

[Region as third level? 6](#_Toc184684549)

[Level two variance visualized 6](#_Toc184684550)

[Client level predictors 8](#_Toc184684551)

[*Crude estimates* 8](#_Toc184684552)

[*Adjusted estimates* 10](#_Toc184684553)

[Facility level predictors 10](#_Toc184684554)

[*Crude estimates* 10](#_Toc184684555)

[*Adjusted estimates* 12](#_Toc184684556)

[Final model 12](#_Toc184684557)

##

## Background

This is a supplementary material for the paper titled *‘A multilevel analysis on the predictors of client satisfaction with family planning services in Ethiopia: Evidence from the Ethiopian Service Provision Assessment (ESPA) 2021/22’.* It will explore the steps taken during the analysis and reports additional information that might be of interest for the reader.

The data was accessed through the [demographic and health survey website](https://dhsprogram.com/publications/publication-spa36-spa-final-reports.cfm?cssearch=1639902_1).

For this research article, two data sets (containing 3 questionnaire types) were merged; namely the client data-set *(containing client-provider observations and client exit interviews)* and the facility data-set *(containing facility inventory data)*. This are named ‘*fpclient’* and’*fpfacility’* respectively.

We used a left_join with *inv_id* as unique identifier so the unit of analysis remained as the *number of clients*.

alldata <- fpclient %>%
 left_join(fpfacility, by='inv_id')

The variables included in our analysis are described in detail as an appendix in the paper.

## Composite measure for client satisfaction

We initially created a composite variable to measure client satisfaction. This composite variable was constructed using *survey question number 202* (refer to the final SPA report), which inquiries about common problems clients face at health facilities.

attach(alldata)
overall_client_satisfaction <- data.frame(c502a,c502b,c502c,c502e,
 c502f,c502g,c502h,c502i,c502j,c502k,
 c502l,c_overall_satisfaction)
overall_client_satisfaction[overall_client_satisfaction == 8] <- NA
overall_client_satisfaction[overall_client_satisfaction == 9] <- NA
overall_client_satisfaction <- na.omit(overall_client_satisfaction)

composite_client_satisfaction <- overall_client_satisfaction %>%
 select(c502a, c502b, c502c, c502e, c502f, c502g, c502h, c502i, c502j, c502k, c502l)

pca_composite <- prcomp(composite_client_satisfaction, scale. = TRUE)
pc1 <- pca_composite$x[, 1]

library(factoextra)
fviz_eig(pca_composite, addlabels = TRUE, main = "Variance Explained by Principal Components")


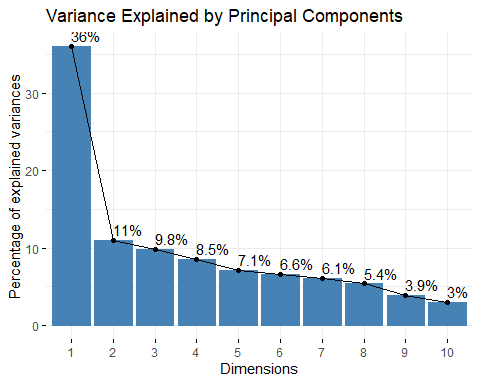


However, once the principal components of the composite measure were identified, a spearman correlation test was conducted to see if the composite variable is actually measuring in line with the overall client satisfaction.

single_variable <- overall_client_satisfaction %>%
 pull(c_overall_satisfaction)

if (length(pc1) == length(single_variable)) {
 cor_spearman <- cor(pc1, single_variable, method = "spearman")
 print(paste("Spearman Rank Correlation: ", cor_spearman))
} else {
 stop("Dimensions of PC1 and c_overall_satisfaction do not match!")
}

## [1] "Spearman Rank Correlation: 0.269412558848388"

## Single variable measure for client satisfaction

The very low correlation between the composite measure and the overall client satisfaction indicates the composite variable is measuring a dimension of patient service that is not correlated with client satisfaction. As a result, a decision to use the single variable measure was made.

In our model client satisfaction is measured using a 4-level likert scale that has been dicothomized into *more satisfied* and *less satisfied*.

## Weights

#Creating facility weight
weight_list <- data.frame(final_df1$f_weight, final_df1$inv_id)
weight_list <- unique(weight_list)
facility_list <- data.frame(final_df1$f_number, final_df1$inv_id)
facility_list<- unique(facility_list)
weight_facility <- weight_list %>%
 inner_join(facility_list, by='final_df1.inv_id')
n1 <- nrow(weight_facility)
weight_facility$final_df1.f_weight <- n1 * (weight_facility$final_df1.f_weight /1000000) /
 sum(weight_facility$final_df1.f_weight /1000000)
sum(weight_facility$final_df1.f_weight)

## [1] 529

# Creating client-weight
n <- nrow(final_df1)
final_df1$c_houseweight <- n * (final_df1$weight/1000000) / sum(final_df1$weight/1000000)
sum(final_df1$c_houseweight)

## [1] 2071

A descriptive table before and after applying weights is presented in the paper.

## Multilevel modelling

## Facility as second level

nullmodel_adapt <- mixed_model(fixed = c_overall_satisfaction ~1 ,
 random = ~ 1 |f_number,
 data = final_df1,
 family = binomial("logit"),
 weights = weight_facility$final_df1.f_weight)

summary(nullmodel_adapt)

##
## Call:
## mixed_model(fixed = c_overall_satisfaction ~ 1, random = ~1 |
## f_number, data = final_df1, family = binomial("logit"), weights = weight_facility$final_df1.f_weight)
##
## Data Descriptives:
## Number of Observations: 2071
## Number of Groups: 529
##
## Model:
## family: binomial
## link: logit
##
## Fit statistics:
## log.Lik AIC BIC
## -963.5288 1931.058 1939.6
##
## Random effects covariance matrix:
## StdDev
## (Intercept) 2.0497
##
## Fixed effects:
## Estimate Std.Err z-value p-value
## (Intercept) 0.3539 0.1213 2.9166 0.003539
##
## Integration:
## method: adaptive Gauss-Hermite quadrature rule
## quadrature points: 11
##
## Optimization:
## method: hybrid EM and quasi-Newton
## converged: TRUE


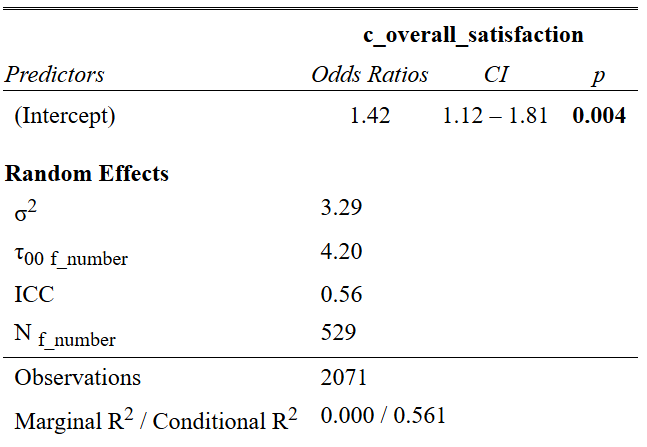


#Median odds ratio
var.area.mnull <- nullmodel_adapt$D
or.mnull <- exp(0.95*sqrt(var.area.mnull))
or.mnull

## (Intercept)
## (Intercept) 7.00914
## attr(,"L")
## [1] 2.0497

## Region as third level?

Conducting a similar test for the variable ‘region’ gives the following result.


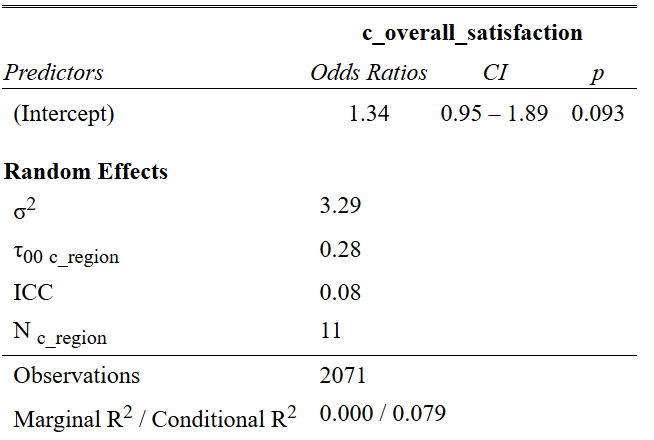


Non significant variance attribution value for region renders it’s use as a third level irrelevant.

## Level two variance visualized

library(ggplot2)
library(egg)
# ggplot butterfly
re = data.frame(condval = unlist(nullmodel_adapt$post_modes)[,1],
 condsd = sqrt(unlist(nullmodel_adapt$post_vars)),
 grp = 1:length(nullmodel_adapt$post_vars))

# sort for plot
dataPlot = re[order(re$condval), ]
dataPlot$rank = 1:NROW(dataPlot)

dataPlot$li = dataPlot$condval - dataPlot$condsd*qnorm(0.975)
dataPlot$ui = dataPlot$condval + dataPlot$condsd*qnorm(0.975)

ggplot(data = dataPlot, mapping = aes(x = rank, y = condval,
 ymin = li,
 ymax = ui))+
 geom_ribbon(fill = "steelblue", color = NA)+
 geom_line()+
 egg::theme_article()


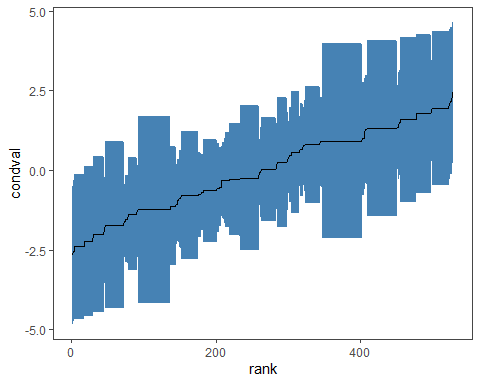


#less dense plot

ggplot(data = dataPlot[as.numeric(as.character(dataPlot$grp)) < 50,],
 mapping = aes(x = rank,
 y = condval, ymin = li, ymax = ui))+
 ylab("Random effects for facility intercept")+
 xlab("Facility rank")+
 geom_hline(color = "pink", yintercept = 0, linetype = "dashed", alpha
 = 0.5)+
 geom_errorbar(color = "steelblue", width = 5)+
 geom_line(color = "steelblue", alpha = 0.1)+
 geom_point(color = "steelblue")+
 egg::theme_article()


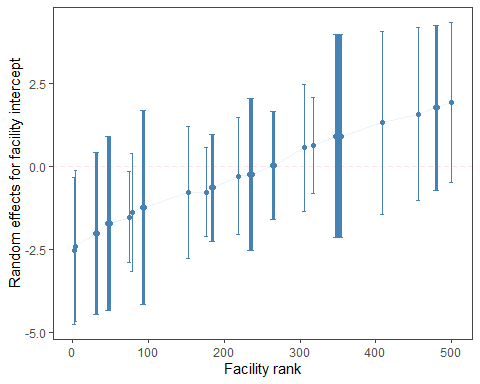


## Client level predictors

## *Crude estimates*

| Variable | Level | OR | LowerCI | UpperCI | p.value |
| --- | --- | --- | --- | --- | --- |
| c_previous_contact | Yes | 1.211265 | 0.846452 | 1.733309 | 0.294515 |
| c_visual_aids | Yes | 1.149979 | 0.720010 | 1.836712 | 0.558573 |
| c_confidentiality | Yes | 1.221142 | 0.763078 | 1.954176 | 0.404939 |
| c_auditory_privacy | Yes | 1.606734 | 1.089872 | 2.368712 | 0.016639 |
| c_visual_privacy | Yes | 1.659904 | 1.063111 | 2.591717 | 0.025799 |
| c_facility_closeness | Yes | 1.398579 | 0.839418 | 2.330214 | 0.197767 |
| c_prepayment_plan | Yes | 0.968116 | 0.691526 | 1.355334 | 0.850277 |
| c_fp_status | Past User | 1.838433 | 1.143491 | 2.955716 | 0.011954 |
| c_fp_status | Never Used | 1.052612 | 0.613309 | 1.806579 | 0.852402 |
| c_wait_time | wait_time | 0.991934 | 0.986219 | 0.997682 | 0.006012 |
| c_asked_ques | Yes | 0.5763606 | 0.4070327 | 0.8161299 | 0.0019035 |
| c_concerns_disscussed | Yes | 0.6921383 | 0.4877266 | 0.9822213 | 0.0393558 |
| c_partner_attitude | Yes | 0.9381833 | 0.4988424 | 1.7644609 | 0.8430454 |
| c_followup | Yes | 1.1020399 | 0.4545849 | 2.6716506 | 0.8297236 |
| c_problem_occur | Yes | 2.6360889 | 1.8987786 | 3.6597025 | 0.0000000 |
| c_sideeffect | Yes | 1.9166866 | 1.3884664 | 2.6458598 | 0.0000764 |
| c_how_to_use | Yes | 1.2376985 | 0.8479216 | 1.8066502 | 0.2691101 |
| c_educationlevel | Primary | 0.5224971 | 0.3678555 | 0.7421480 | 0.0002884 |
| c_educationlevel | Higher | 0.6390332 | 0.4312163 | 0.9470039 | 0.0256594 |
| c_maritalstatus | Married | 0.9940164 | 0.5220273 | 1.8927526 | 0.9854277 |
| c_age | 22 TO 30 | 1.3739005 | 0.9764225 | 1.9331823 | 0.0682937 |
| c_age | > 30 | 3.5774911 | 2.2794761 | 5.6146421 | 0.0000000 |

## *Adjusted estimates*

Adjusted estimates along with LLR, median odds ratio and proportional change in variance are reported in the original paper.

##

## Facility level predictors

## *Crude estimates*

| Variable | Level | OR | Low.CI | Upp.CI | p.value |
| --- | --- | --- | --- | --- | --- |
| f_training | Yes | 0.553223 | 0.333073 | 0.918885 | 0.022209 |
| f_catchment_pop | 25K TO 1.5M | 1.080917 | 0.640113 | 1.825274 | 0.770983 |
| f_catchment_pop | More than 1.5M | 2.829981 | 1.214277 | 6.595522 | 0.015964 |
| f_no_of_days | Above 20 | 1.187036 | 0.721410 | 1.953192 | 0.499795 |
| f_quality_structure | Quality Unit & Committe | 2.442225 | 1.222477 | 4.87899 | 0.011440 |
| f_quality_structure | Quality Committe | 3.11022 | 1.909801 | 5.065187 | 0.000005 |
| f_guideline_national | Available | 1.222454 | 0.75815 | 1.971094 | 0.409892 |
| f_routine_action | Yes | 0.910758 | 0.567235 | 1.462322 | 0.698804 |
| f_location | Rural | 0.477084 | 0.292322 | 0.778626 | 0.003064 |
| f_ownership | Private | 2.390143 | 1.049041 | 5.445719 | 0.038083 |
| f_no_trained_provider | Yes | 0.65007 | 0.390870 | 1.081170 | 0.097054 |
| f_methods_offered | >= 8 | 0.959998 | 0.591529 | 1.557989 | 0.868751 |
| f_supervisory_visit | Within 6 month | 1.415565 | 0.597381 | 3.354349 | 0.429797 |
| f_supervisory_visit | More than 6 month | 1.032635 | 0.534270 | 1.995875 | 0.923902 |
| f_routine_fees | Fixed | 0.365071 | 0.16495 | 0.807956 | 0.012914 |
| f_routine_fees | Separate | 2.477593 | 1.526982 | 4.019997 | 0.000238 |
| f_monthly_meetings | Yes | 0.712344 | 0.405315 | 1.25195 | 0.2384079 |
| f_comm_meetings | Yes | 0.851993 | 0.527484 | 1.376139 | 0.512601 |
| f_waiting_area | Yes | 4.968715 | 2.612599 | 9.449643 | 0.000001 |
| f_standard_precaution | Yes | 1.59904 | 0.973885 | 2.62556 | 0.063538 |
| f_performance_check | Clinical audit | 1.393650 | 0.725050 | 2.678794 | 0.319436 |
| f_performance_check | Regular audit | 1.158927 | 0.663852 | 2.023210 | 0.603876 |
| f_performance_check | Feedback | 3.158816 | 1.441026 | 6.924312 | 0.004073 |
| f_dhis2 | Yes | 1.70492 | 1.062657 | 2.735363 | 0.026969 |
| f_risk_assessment | Yes | 1.044942 | 0.601979 | 1.813857 | 0.875844 |

## *Adjusted estimates*

Adjusted estimates along with LLR, median odds ratio and proportional change in variance are reported in the original paper.

## Final model

A combination of client and facility level variables that were significant in the previous models are reported for adjusted estimates along with LLR, median odds ratio and proportional change in variance in the original paper.

*Thank you for taking an interest in our work.*
